# Immediate Outcomes and Benefits of 3D Printed Braces for the Treatment of Adolescent Idiopathic Scoliosis

**DOI:** 10.3389/fresc.2022.840286

**Published:** 2022-02-28

**Authors:** Edmond Lou, Kenwick Ng, Doug Hill

**Affiliations:** ^1^Department of Electrical and Computer Engineering, University of Alberta, Edmonton, AB, Canada; ^2^Department of Biomedical Engineering, University of Alberta, Edmonton, AB, Canada; ^3^Research Innovation and Technology, Glenrose Rehabilitation Hospital, Edmonton, AB, Canada

**Keywords:** brace treatment, adolescent idiopathic scoliosis, ultrasound assisted casting, spinal flexibility, in-brace correction, three-dimensional printing

## Abstract

Spinal bracing is a proven effective treatment for children with adolescent idiopathic scoliosis (AIS). Four factors have been reported to affect brace treatment outcome including (1) growth or curve-based risk, (2) the in-brace correction, (3) the brace wear quantity, and (4) the brace wear quality. The in-brace correction is impacted by spinal flexibility. The quality of brace design also affects the in-brace correction and comfort which indirectly affects the brace wear quantity and quality. A traditional polypropylene spinal brace is bulky and uncomfortable, and its manufacturing process is labor intensive. As 3D printing technology becomes more common and advanced, there is a potential to manufacture spinal braces using 3D printing technology. The objectives of this paper were to report the immediate effectiveness and benefits in using 3D printed brace to treat children with AIS. Six children with AIS (5F, 1M; 12.9 ± 1.4 years old; Cobb angle: 26° ± 7°), who were new to brace treatment, were recruited. Spinal flexibility and pressure pad locations were acquired using ultrasound assisted method to ensure braces were designed properly. To manufacture the braces, all participants were scanned by a handheld 3D scanner to obtain their body shapes. The 3D braces were then printed with Nylon 12 material. The average in-brace Cobb angle correction was 10 ± 4° (41 ± 18% correction). The 3D brace was 33% thinner, 26% lighter, 37% lower cost and required 3.7 h less labor time to manufacture when compared with the standard polypropylene brace. As a conclusion, the 3D printed brace had good immediate treatment effectiveness, but the long-time effect is still required time to explore.

## Introduction

Adolescent Idiopathic Scoliosis (AIS) is a three-dimensional (3D) spinal disorder with a lateral curvature and axial vertebral rotation (AVR) (1). It affects 1–3% of adolescents (1, 2). The Cobb angle measured on a posteroanterior (PA) radiograph is the gold standard to assess its severity and evaluate treatment outcome. Treatment modalities for AIS are based on consideration of the patient's physiologic (not chronologic) maturity, curve severity, curve location and the estimated risk of progression (3). Brace treatment is the most commonly used non-surgical treatment for scoliosis, and its goal is to prevent further curve progression. To be effective, the brace is recommended to be worn properly for up to 23 h per day until the child has completed growth (4). Weinstein et al. multicenter randomized clinical trial reported that bracing significantly decreased the progression of high-risk curves (5). A full-time brace patient who wore the brace for an average of at least 12.9 h/day was associated with a high success rate. However, limitations of this multicenter study were the unknown quality of brace wear and the spinal flexibility information. Predicting brace treatment outcomes had been reported using both quantity and quality of brace wear and the in-brace correction (6). He et al. also reported that the immediate in-brace correction was correlated with spinal flexibility (7). Khodaei et al. demonstrated that the most reliable method to estimate spinal flexibility on non-surgical candidates using ultrasound images was to use the bending relative to standing index (BRSI) (8).

A conventional spinal brace is a rigid thermoplastic jacket with multiple foam pads added inside the inner surface to provide directed mechanical loads on the torso to counteract the spinal curvatures. The manual design process can be cumbersome, labor intensive and costly, and requires applying wet plaster wraps on the patient torso to obtain a negative body mold. The body mold is then scanned by a 3D handheld scanner. A 3D torso file is then generated and can be sent to a carver machine to create a positive foam mold. A polypropylene sheet is then thermo-vacuum formed on the foam mold to create the brace shape. Custom trimming the brace edges and adding brace accessories are required before fitting the brace to patient. With the advancement of 3D scanning and printing technologies, a computer-aided design/computer-aided manufacturing (CAD/CAM) system can capture the torso contour using a 3D handheld scanner directly. The scanned torso file is then modified and sent to a 3D printer for manufacturing a brace. Pilot studies had been conducted in developing 3D printed scoliosis braces (9–13, 15) and exploring treatment effectiveness and in-brace comfort (14). Zhang et al. (9) developed a topology optimization technique for 3D printing which can be used to manufacture spinal orthosis. Although they reported that their spinal orthosis could reduce 50–70% printing material, validation was only performed on finite element simulation. No clinical trial was conducted. A research team in China also conducted a feasibility study (10) to validate using 3D printing technology to manufacture spinal orthosis. They did a single case and reported a 50% in brace correction. They also mentioned that more cases and long-term follow-up are required. Weiss et al. (11) provided a detailed workflow of the current CAD/CAM—carving method to produce a spinal orthosis. They also provided an outline for future requirements with respect to 3D printing technology. Shah and Luximon (12) reported the process on how to manipulate the human body shape file and combine it with finite element analysis to design a 3D spinal orthosis. They concluded that 3D printed spinal orthosis could be easily manufactured in the near future. Recently, a RCT study (15) to evaluate the clinical effectiveness of 3D-printed orthosis was reported. Their orthoses design was based on the orthosis judgment without using ultrasound or radiography for objective assessment. They also used an expensive 3D printing machine (FORTUS 900MCTM, Stratasys Ltd, Eden Prairie, USA). Its cost is comparable to a carving machine (4-Axis Carving Machine, Vorum, Canada). Their results also demonstrated that 3D printed orthosis could provide equivalent treatment effects as the conventional orthosis for the patients with AIS.

Among many 3D printing methods, fused deposition modeling (FDM) method is the most suitable approach. Furthermore, there are many materials which are suitable and compatible with the traditional polypropylene brace material. Ng et al. (13) recommended Nylon12 while Redalli et al. (14) suggested Polyethylene terephthalate glycol- modified (PETG) material. The Nylon12 material with 2.5 mm thickness had 44.1% lower stiffness, 8.6% lower break force and 34.4% higher yield force than the standard brace material. Furthermore, the force stroke-displacement curves of 2.5 mm and 3.25 mm Nylon12 provided close characteristics to polypropylene. However, the reported 3D printed brace studies remain limited. The objectives of this study were to investigate the immediate 3D printed brace treatment effectiveness and to evaluate the manufacturing process when compared to a standard scoliosis brace process.

## Materials and Methods

### Study Participants

Participants who were (a) diagnosed with AIS, (b) age between 10 and 16 years old, (c) pre-menarchal or <1 year post-menarchal for female, and had (d) pre-brace largest Cobb angle between 20 and 45°, (e) Risser sign <3, and (f) prescribed with a full time (23 h) thoracic-lumbar-sacral orthosis (TLSO) were recruited from the local scoliosis clinic. Ethics approval was granted from the local health research ethics board, and all participants and their guardians signed the assents and parental consent forms prior to participation.

### Spinal Flexibility

To assess the spinal flexibility, two or three ultrasound (US) images were acquired from each participant. The US scans were acquired at standing, maximal prone left or/and right side bending. The direction of side bending depended on whether the subject had the left-side or right-side curve, bending to the opposite side of the curve. An experienced US operator performed all US scans during this study. All scans started from the vertebral level C7 and terminated at L5 with both levels were marked before scanning. During scanning, the US probe was positioned perpendicular to the participants' back and moved along the path of the curve. For standing position, subjects were asked to stand in a standard upright posture. In maximal side bending, participants were instructed to bend their upper body as much as they could to the left or right side without moving their pelvis. The spinal flexibility index, bending relative to standing index (BRSI), was calculated using Equation (1):


(1) $$\text{BRSI }=\frac{\text{US standing Cobb}-\text{ US Bending Cobb}}{\text{US standing cobb}}$$

More flexible curves have higher BRSI values. A BSRI >1.0 implies overcorrection.

### Optimization of Brace Pad Location and Pressure Levels

**Figure 1:**
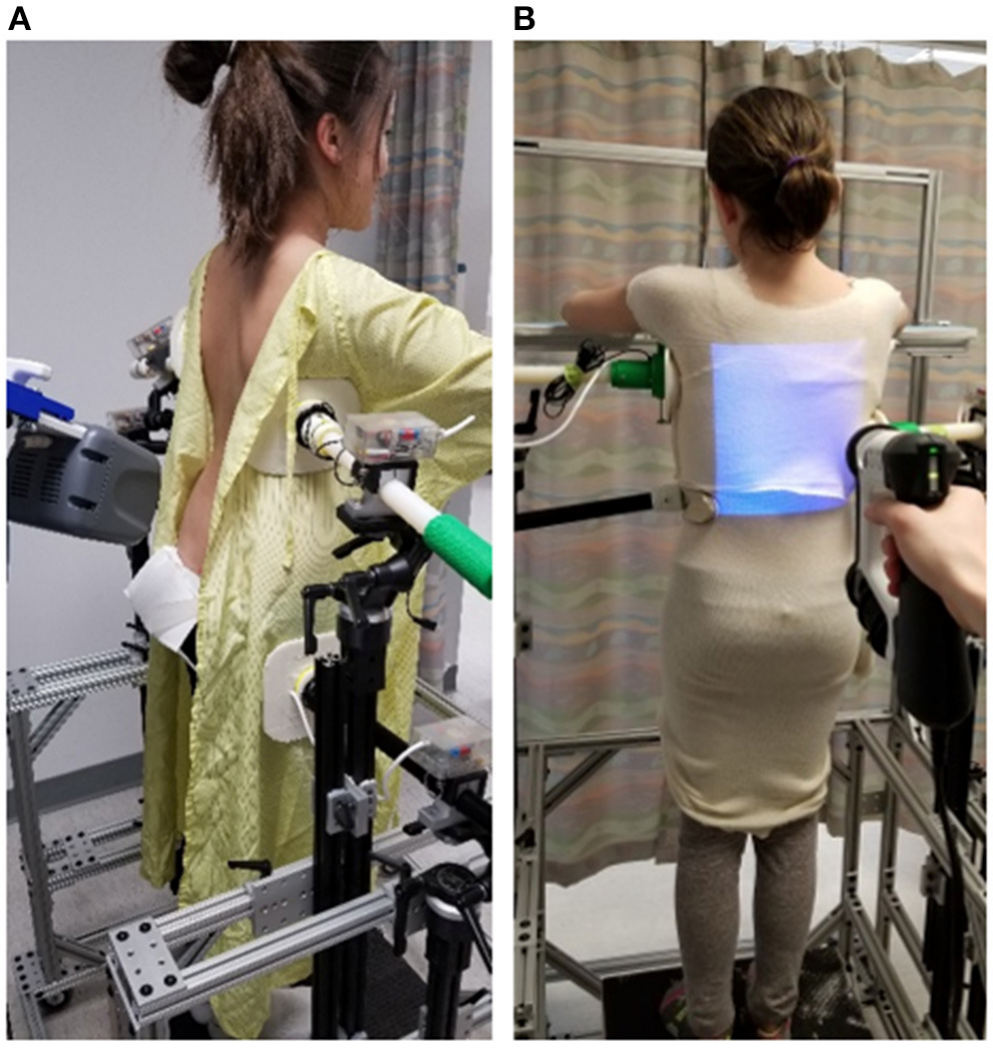
A participant standing inside a custom frame **(A)** for ultrasound assisted brace casting, **(B)** for body scan using a 3D Spectra Scanner.

Before designing the brace, the pre-brace x-ray and the standing US spinal image were displayed side-by-side to assist the orthotist to decide on pressure pad locations. Participants were asked to don a gown with the back opened and stood inside a custom 3D brace design frame. The orthotist used the design frame to secure bolsters with subjectively determined applied pressure levels against the patient's torso to simulate in-brace correction. Figure 1A shows a participant standing inside a frame with bolsters applied to the body. An air bag was attached to the surface of each bolster to measure the interface pressure applied between the bolster and body. The simulated in-brace US scan was then acquired. The simulated in-brace Cobb angle was measured on the US image using in-house developed software. This process took <1 min. This US assisted method had been demonstrated to provide a radiation-free method to determine the optimum pressure level and location to assist brace design, resulting in decreased radiation exposure during follow-up brace evaluation, increased the in-brace correction, reduced the patients' visits to both brace adjustment and scoliosis clinics (16, 17). The orthotist then decided if altering bolster locations and orientations and pressure levels might improve correction. Another US scan was taken if the bolster positions or pressure level were altered. The procedures were repeated until the orthotist attained the best simulated in-brace correction configuration. The target goal was try to get ~50% correction when the spinal flexibility BSRI was around 1. During scanning, the pressure levels at each bolster were recorded.

### 3D Printed Brace

**Figure 2:**
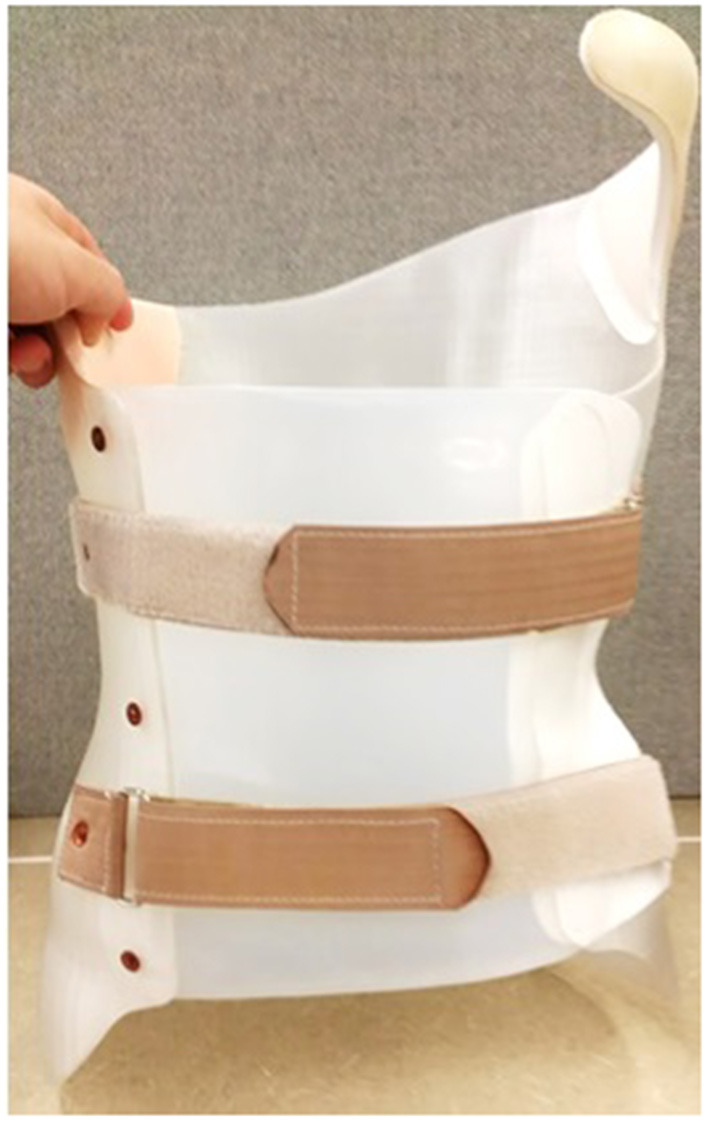
An anterior opening of a 3D printed Nylon12 brace.

After determining the optimal pad location and pressure level, participants were asked to put on a stocking shirt and stand at the same position. A 3D Spectra scanner (Vorum, Vancouver, Canada) was used to capture the 3D torso as shown in Figure 1B. After the body file was obtained, the orthotist modified the scanned body contour by virtually adding pressure and relief regions, sanding and smoothing, and outlining brace shape. The braces shape file was further modified with Meshmixer software (Autodesk, California, USA) for compatibility with 3D printing. This included trimming the brace shape according to the orthotist's outline, adding overall and additional thickness at regions that required greater strength or stiffness, as well as generating supporting structures for 3D printing. All braces were printed with a BIG-60 (Modix, Tel-Aviv, Israel) 3D printer using Nylon12 material in 2.5 mm design thickness which was based on the Ng et al. study (13). Printing process parameters were adjusted with Cura software (Ultimaker, Utrecht, Netherlands). Post-processing involved support material removal and soaking the printed brace in a heated water bath to improve mechanical properties. Figure 2 shows a finished 3D printed brace. In addition to the 3D printed brace, a traditional polypropylene brace was manufactured for each participant in case of failure of the 3D printed braces during manufacturing or wear.

### Outcome Measures

All participants returned to the scoliosis clinics ~6 weeks after they started using their braces. In-brace radiographs were taken at the first follow-up visit using an EOS radiography system (EOS Imaging S.A., Paris, France). The in-brace Cobb angle was measured by an experienced rater who had over 20 years of experience.

Brace production time (casting, fitting, labor, machine and post-processing) was recorded. Mass ratio between the Nylon12 printed (m_*N*_) brace and a polypropylene (m_*pp*_*)* brace was calculated using Equation (2):


(2) $$\begin{array}{r} \frac{m_{N∙}}{m_{pp∙}}=\frac{\rho_{N}}{\rho_{pp}}x\frac{t_{N}}{tpp}x\frac{H_{N}{∙L}_{N}}{H_{pp∙}L_{pp}} \end{array}$$

where m is mass, ρ is density (kg/m^3^), t is the thickness of plastic sheet (m), H is the height of plastic sheet (m), and L is the length of plastic sheet (m). Both traditional and 3D braces were estimated based on a rectangular volume of a polypropylene sheet.

Cost analysis included material, labor cost, and equipment cost. Labor cost included orthotist (CAD $100/h) and technician costs (CAD $50/h). Equipment cost included the foam mold carver and the 3D printer.

## Results

**Figure 3:**
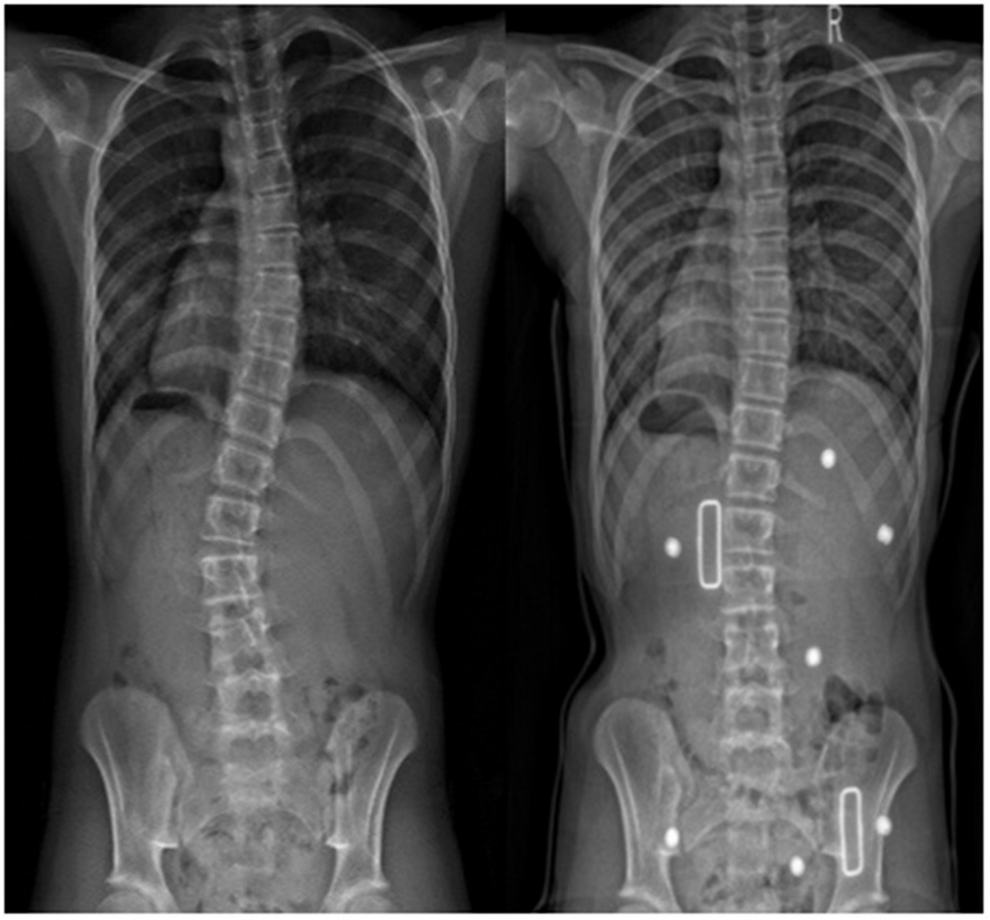
Pre-brace (left) and in-brace radiographs (right) of a participant.

Six participants, 5 females and 1 male, age ranged between 11.4 and 14.9 years, were recruited. All 3D printed braces were well-manufactured and none of the participants were required to use the traditional backup braces. Table 1 summarizes the pre-brace characteristics including gender, Risser grade, age, BMI, curve type, and the calculated spinal flexibility based on equation (1). Most of the participants (5/6) were immature with the Risser sign below 2. The average BRSI for thoracic and thoracolumbar/lumbar major curves were 1.25 and 1.4 ± 0.2, respectively; all were >1 with the major treated curves very flexible and able to overcorrect during the maximum bending test. The average major Cobb angle at pre-brace and in-brace was 26° ± 7° (range 20°-36°) and 16° ± 8° (range 5°-27°), respectively. The average correction was 10° ± 4° (41 ± 18%). Table 2 summaries the pre-brace and in-brace correction information. Figure 3 shows PA radiographs of pre-brace and in-brace from one of the participants.

**Table 1:** Participants' baseline characteristics.

Pre-brace characteristics	Gender	1M: 5F
	Risser: 0 and 1	5/6 (83%)
	Risser: 2	1/6 (17%)
	Age (years)	12.9 ± 1.4
	BMI^a^	20.2 ± 3.3 (16.0–23.6)
Pre-brace curve classification	Number of treated curves	6
	Thoracic flexibility (BRSI) (*n* = 2)	1.26 (1.25–1.26)
	Thoracolumbar/lumbar flexibility (BRSI) (*n* = 4)	1.4 ± 0.2 (1.19–1.72)

a *BMI, Body mass index is calculated based on body weight and height*.

**Table 2:** Pre-brace and in-brace curve characteristics.

**Curve type**	**Initial cobb**	**Simulated in-brace cobb**	**Simulated % correction**	**In-brace cobb**	**In-brace correction**	**BRSI**
LL	28	16	42.9	15	13	1.72
RT	20	11	45.0	11	9	1.25
LL	22	13	40.9	16	6	1.2
LL	31	20	35.5	24	7	1.32
RT	20	5	75.0	5	15	1.26
LL	36	25	30.6	27	9	1.19
Mean	26.2	15.0	45.0	16.3	9.8	1.3
SD	6.6	7.0	15.6	8.1	3.5	0.2

*LL, Left Lumbar; RT, Right Thoracic*.

**Figure 4:**
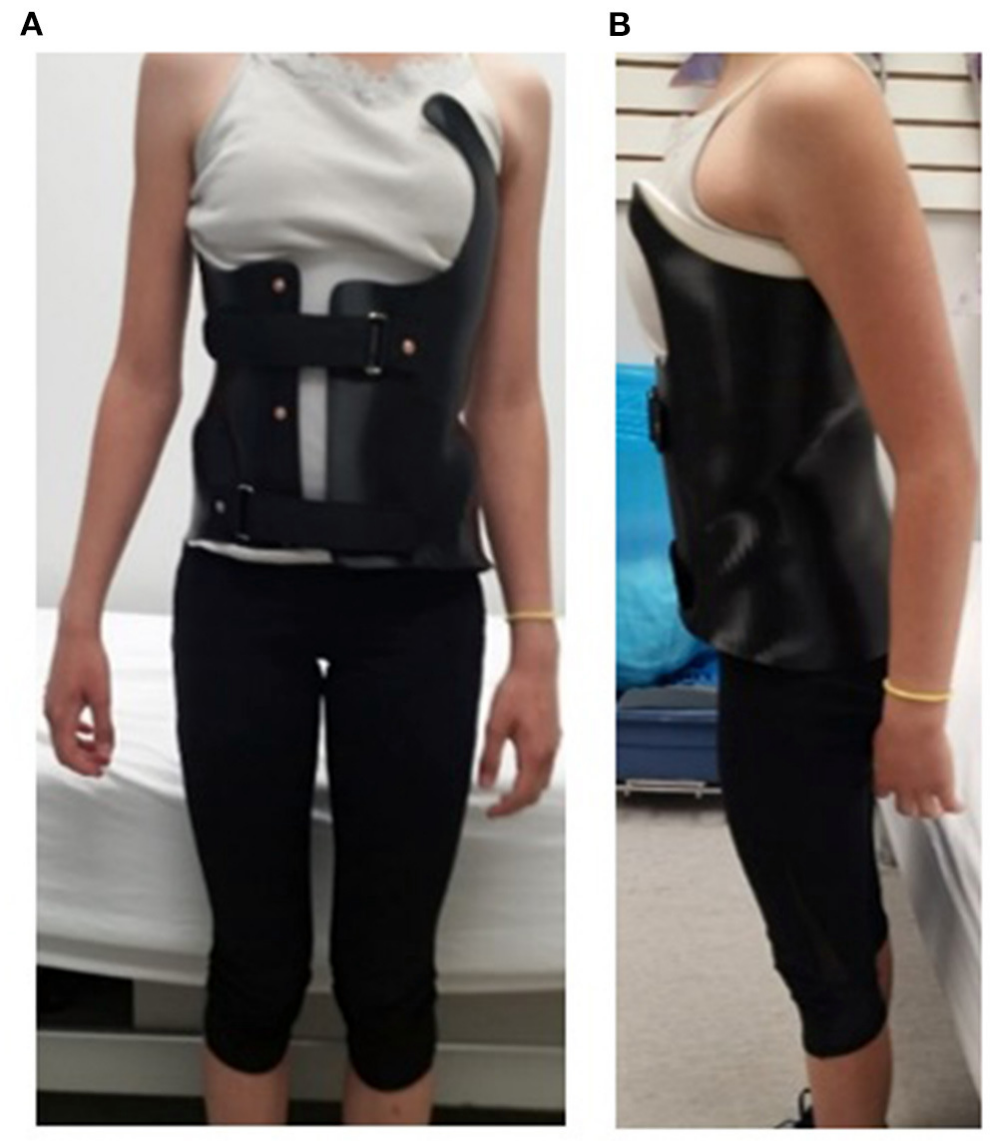
A participant wearing a 3D printed Nylon12 brace in **(A)** frontal view, and **(B)** side view.

Table 3 summarizes the brace casting, fitting and manufacturing time, as well as the brace characteristics for the traditional polypropylene and 3D printed braces. A difference of 0.4 h of casting and fitting time was observed. Regarding the manufacturing time, thermo-vacuum forming was the most time-consuming process on the polypropylene brace. The total labor time for the traditional brace vs. the 3D printed brace was 6.25 vs. 2.58 h (3.7 h difference). However, the polypropylene brace was ready for use in 6.75 h, but the 3D printed brace required 58 h to being ready for use. This time includes 31 h printing time and 24 h water treatment time. Water treatment improved the mechanical property of the nylon12 brace. The thickness of a traditional brace was 4.5 mm compared to printed thickness of 3 mm for 3D brace (even though designed thickness was 2.5 mm). Figure 4 shows a participant fitting her 3D printed brace. The weight ratio between a traditional and a 3D printed brace was 1:0.74. The total cost of a traditional vs. 3D printed brace was CAD $563 vs. CAD $356. The most significant fixed cost was the initial equipment, which was CAD $200K for the carver machine and CAD $10K for the 3D printer.

**Table 3:** Comparisons of time, thickness, weight, and cost between traditional and 3D printed brace.

	**Traditional brace**		**3D printed brace**	
	**Components**	**Time (min)**	**Components**	**Time (min)**
(A) Casting and fitting time (Orthotist time)	(1) Ultrasound assisted brace casting	45	(1) Ultrasound assisted brace casting	45
	(2) Casting time: body wrap	30	(2) Direct body scan	5
	(3) Follow-up fitting clinic for brace adjustment	15	(3) Follow-up fitting clinic for brace adjustment	15
	Subtotal	90 (1.5 h)	Subtotal	65 (1.1 h)
(B) Brace manufacturing labor time (Technician time)	(1) Scan the body mold^*^	30		
	(2) 3D body mold model modification	30	(1) 3D body model modification	45
	(3) Set up foam mold carver^*^	15	(2) Set up 3D printer -print settings^*^	30
	(4) Thermoform and trim brace, add brace accessories^*^	210 (3.5 h)	(3) Surface finish, add brace accessories^*^	15
	Subtotal	285 (4.75 h)	Subtotal	90 (1.5 h)
(C) Machine and post-processing time	(1) Carve positive mold	30	(1) 3D brace printing	1,860 (31 h)
			(2) Water treatment	1,440 (24 h)
	Subtotal	30	Subtotal	3,300 (55 h)
Total labor time (h) (A) + (B)		6.25	Total labor time (h)	2.58
Total time (h) (A) + (B) + (C)		6.75	Total time (h)	57.58
	Traditional brace		3D printed brace	
Thickness (mm)	4.5		3.0	
Weight ratio	1		0.74	
Material cost (CAD$)	150		135	
Labor time	Total orthotist time = 120 min Total technician time = 255 min		Total orthotist time = 110 min Total technician time = 45 min	
Labor cost (CAD$)	2^*^100 + 4.25^*^50 = 412.5		1.83^*^100 + 0.75^*^45 = 220.8	
Equipment cost (CAD$)	200K		10K	

* *Indicates technician labor hours*.

## Discussion

In current practice, a skilled and an experienced orthotist can design high quality of spinal braces. During designing a brace, it is necessary to compromise between the comfort and treatment outcomes. The comfort level is influenced by how aggressively the orthotist designs the brace. On the other hand, the prediction of the treatment outcome is usually based on the immediate in-brace correction. Without an objective evaluation of spinal flexibility, clinicians aim to achieve 40–50% in-brace Cobb correction (18–20). If the in-brace correction is not deemed to be satisfactory by the treating orthopedic surgeon, the patient returns to the orthotist for readjustment. This adjustment increases cumulative radiation exposure and shortens effective brace usage. The strength of this study was the spinal flexibility was objectively evaluated. This allowed the orthotist to estimate the intended in-brace correction for the patient during simulated in-brace condition. Another strength was the inclusion of ultrasound assisted brace casting. This provided quantitative applied pressures and real-time simulated in-brace correction for the orthotist to assess the brace design. Literature had shown that fewer brace adjustments clinic visits were required with ultrasound assisted brace casting (16, 17).

In term of manufacturing process, the total 3D brace labor time was 3.7 h shorter than the polypropylene brace. Although the actual manufacturing time of the 3D brace was 8.5 times longer, most of the time was the machine and post-processing time (96%) rather than labor time. Machine and post-processing could be done overnight and outside of normal work hours. The reduced labor time not only lower health care cost, but also reduced labor effort which allowed clinicians to provide more time for direct patient contact. The 3D printed brace was 33% thinner and 26% lighter. A thinner and lighter brace will be less noticeable, dissipate heat better, and may thus improve brace wear compliance.

Comparing the materials and methods of manufacturing with other studies, Redaelli et al. (14) used the Structure Sensor and Autodesk Meshmixer software (Autodesk, California, USA) for body shape capture and 3D printing file preparation. They used PETG material with 2.2 mm thickness to print a brace. Based on the material property of PETG, the printing surface of PETG is usually rough. Post processing may be required to smooth the surface finish. Zhang and Kwok (9) used polycarbonate and printed with 4 mm thickness. Polycarbonate is a strong and stiff material, with slower heat conduction. The Nylon12 material used in this study has been extensively evaluated (13). It was modifiable, flexible and cost effective and the Nylon12 brace was able to handle 2,920 times simulated open and close procedures (4x/day for 2 years) and still providing similar strength and flexibility characteristics as the standard brace.

Two of the participants were randomly selected and asked to compare the 3D printed brace and the traditional brace. Both wore their prepared traditional brace for a day. Having experience to try both types of braces, both participants preferred the 3D printed braces. They both reported that the 3D printed brace was more comfort to wear because of being thinner and lighter.

The limitation of this study is the number of participants is small, and the long-term effectiveness is unknown. It also requires an experienced ultrasound technician during the brace casting to acquire and analyze the data. To overcome this, an automatic ultrasound machine which can scan the back automatically is being considered for future improvements. Also, the custom software developed for the ultrasound imaging measurement needs to be enhanced so that 3D information and automatic measurements can be obtained without requiring significant operator experience.

## Conclusions

The 3D printed brace was as effective as the traditional polypropylene brace. The immediate in-brace correction was achieved to the target goal level. The 3D printed brace was 33% thinner, 26% lighter, 37% lower in cost, and required 3.7 h less labor time to manufacture than a standard brace.

## Data Availability Statement

The raw data supporting the conclusions of this article will be made available by the authors, without undue reservation.

## Ethics Statement

The studies involving human participants were reviewed and approved by the Health Research Ethics Board of the University of Alberta. Written informed consent to participate in this study was provided by the participants' legal guardian/next of kin.

## Author Contributions

EL: made substantial contributions to the conception and design of the work, interpretation of data, drafted the work and revised it critically for important intellectual content, approved the version to be published, and agree to be accountable for all aspects of the work in ensuring that questions related to the accuracy or integrity of any part of the work are appropriately investigated and resolved. KN: made substantial contributions to the conception and design of the work, data acquisition, data analysis, interpretation of data, drafted the work, approved the version to be published, and agree to be accountable for all aspects of the work in ensuring that questions related to the accuracy or integrity of any part of the work are appropriately investigated and resolved. DH: made substantial contributions to the conception and design of the work, approved the version to be published, and agree to be accountable for all aspects of the work in ensuring that questions related to the accuracy or integrity of any part of the work are appropriately investigated and resolved. All authors contributed to the article and approved the submitted version.

## Funding

This work was supported by the Women and Children's Health Research Institute and Natural Sciences and Engineering Research Council of Canada.

## Conflict of Interest

The authors declare that the research was conducted in the absence of any commercial or financial relationships that could be construed as a potential conflict of interest.

## Publisher's Note

All claims expressed in this article are solely those of the authors and do not necessarily represent those of their affiliated organizations, or those of the publisher, the editors and the reviewers. Any product that may be evaluated in this article, or claim that may be made by its manufacturer, is not guaranteed or endorsed by the publisher.
